# Clinical and Echocardiographic Findings in an Aged Population of Cavalier King Charles Spaniels

**DOI:** 10.3390/ani11040949

**Published:** 2021-03-28

**Authors:** Jorge Prieto Ramos, Andrea Corda, Simon Swift, Laura Saderi, Gabriel De La Fuente Oliver, Brendan Corcoran, Kim M. Summers, Anne T. French

**Affiliations:** 1 Anicura Uribe Kosta Clinica Veterinaria, 48600 Sopelana, Spain; jorge@medcardiovet.com; 2 Department of Veterinary Medicine, University of Sassari, 07100 Sassari, Italy; 3 College of Veterinary Medicine, University of Florida, Gainesville, FL 32608, USA; sswift@ufl.edu; 4 Department of Medical, Surgical and Experimental Sciences, University of Sassari, 07100 Sassari, Italy; lsaderi@uniss.it; 5 Departamento de Ciencia Animal, Universitat de Lleida-Agrotecnico-CERCA, 25198 Lleida, Spain; gabriel.delafuente@udl.cat; 6 Royal (Dick) School of Veterinary Medicine, Easter Bush Campus, University of Edinburgh, Midlothian EH25 9RG, UK; brendan.corcoran@ed.ac.uk; 7 Translational Research Institute, Mater Research Institute-University of Queensland, 37 Kent St, Wooloongabba, QLD 4102, Australia; kim.summers@mater.uq.edu.au; 8 School of Veterinary Medicine, Ross University, Two mile hill st., Michael BB11093, Barbados; afrench@rossvet.edu.kn

**Keywords:** myxomatous mitral valve disease, echocardiography, mitral valve degeneration, mitral valve prolapse, cardiac remodelling, dog, cardiology, Cavalier King Charles Spaniel

## Abstract

**Simple Summary:**

Myxomatous mitral valve disease is the most common cardiac disease in dogs. Cavalier King Charles Spaniels are particularly susceptible to this disease, which generally appears earlier in life than other breeds, resulting, in some cases, in congestive heart failure and death. We hypothesised that within the elderly Cavalier King Charles Spaniels population, there is a sub-cohort of myxomatous mitral valve disease-affected dogs that do not have chamber enlargement. The objectives of the present study were to determine the prevalence and the degree of cardiac chamber enlargement associated with the disease in a population of aged Cavalier King Charles Spaniels, and to assess the effect of age, gender, and body weight on echocardiographic status. A total of 126 Cavalier King Charles Spaniels aged over eight years old were prospectively included in the study. On echocardiographic examination, 100% of them were diagnosed with myxomatous mitral valve disease; 55.6% of them presented chamber enlargement, and 44.4% did not. Age was significantly associated with the presence and the severity of cardiac chamber enlargement and mitral valve prolapse. Our results showed that a proportion of elderly Cavalier King Charles Spaniels with confirmed myxomatous mitral valve disease did not undergo advanced stages of this pathology.

**Abstract:**

Myxomatous mitral valve disease (MMVD) is the most common cardiac disease in dogs. It varies from dogs without clinical signs to those developing left-sided congestive heart failure, leading to death. Cavalier King Charles Spaniels (CKCSs) are particularly susceptible to MMVD. We hypothesised that within the elderly CKCS population, there is a sub-cohort of MMVD-affected dogs that do not have cardiac remodelling. The objectives of the present study were (i) to determine the prevalence and the degree of cardiac remodelling associated with MMVD; and (ii) assess the effect of age, gender, and body weight on echocardiographic status in a population of aged CKCSs. A total of 126 CKCSs ≥ 8 years old were prospectively included. They all had a physical and echocardiographic examination. A systolic murmur was detected in 89% of dogs; the presence of clinical signs was reported in 19% of them; and echocardiographic evidence of MMVD was described in 100%. Despite the high prevalence, 44.4% of the dogs were clear of echocardiographic signs of cardiac remodelling. Age was significantly associated with the presence and severity of cardiac remodelling and mitral valve prolapse. Our results showed that a proportion of elderly CKCS with confirmed MMVD did not undergo advanced stages of this pathology.

## 1. Introduction 

Myxomatous mitral valve disease (MMVD) is the most common acquired cardiac disease in dogs [1,2,3,4]. In about one-third of cases, the tricuspid valve is also affected [1,5]. The incidence of the disease increases with age, and valve changes can be seen on necropsy in nearly all dogs over the age of nine years [6]. Despite this wide incidence, the natural history varies from dogs that do not show clinical signs, to those developing clinical signs consistent with left-sided congestive heart failure leading to death [3]. Echocardiography is now used routinely to diagnose MMVD and to monitor the degree of cardiac remodelling and the haemodynamic changes associated with mitral regurgitation (MR) and to help predict the outcome [7]. Previous studies have identified echocardiographic markers of disease severity, progression and poor outcome, including left atrial (LA) and left ventricular (LV) enlargement, poor LA and LV function, presence of concurrent pulmonary hypertension (PH), and increased early transmitral flow velocity [8,9,10,11,12]. The extent of cardiac remodelling is important as a marker of disease progression and is also used to inform the starting point for therapeutic intervention [4].

Certain pedigree breeds are recognised to be particularly susceptible to MMVD where the disease appears in early adulthood, progresses over several years, and results in earlier onset of congestive heart failure and death. Cavalier King Charles Spaniels (CKCSs) are particularly noted in this regard; MMVD has been well characterised in the breed [13,14,15,16,17,18]. Prevalence of left apical systolic heart murmurs, suggestive of MMVD, was found in 100% of CKCSs 10 years or older [16,18,19]. However, there are anecdotal reports from breeders and owners of elderly CKCSs that are not adversely affected by the disease, whether reported to be murmur-free or not. Moreover, a study conducted in France reported that 38.5% of CKCSs older than 10 years with echocardiographic evidence of MMVD did not have LA or LV dilation [19].

We hypothesised that within the elderly CKCS population, there is a sub-cohort of MMVD-affected dogs that do not have cardiac remodelling. The objectives of this study were, therefore, to estimate the prevalence and the degree of cardiac remodelling associated with MMVD in a population of aged CKCSs and determine whether there is an effect of age, gender, and body weight (BW) on echocardiographic status.

## 2. Materials and Methods

The study was undertaken at the Royal (Dick) School of Veterinary Studies (R(D)SVS), the University of Edinburgh. Regional CKCS clubs were contacted to distribute information regarding the study and to recruit aged CKCSs for the study. Permission for the use of data was obtained with full owner consent and was approved by the Veterinary Ethics in Research Committee (VERC) and the Human Ethics in Research Committee (HERC) of the R(D)SVS. This was a cross-sectional study in which CKCSs eight years of age or older were prospectively included. The examinations were performed at 13 different locations across the United Kingdom, including breed shows, owner homes, Small Animal Hospital, School of Veterinary Medicine, the University of Glasgow, and at the R(D)SVS. Age under eight years was the only exclusion criterion for entry into this study. A history was taken from each dog, looking for signs suggesting cardiac or concurrent diseases. The age, BW, gender, reproductive status, and any concurrent medication given were also recorded. Each dog had a physical examination performed by a board-certified veterinary cardiologist (A.F. and S.S.). Heart rate (HR) and respiratory rate (RR), presence of change in respiratory rate and/or effort, abdominal distension, jugular pulse, murmur, and any rhythm irregularity were recorded. The timing, location, and intensity of the murmur were also recorded. The murmur intensity was scored using the Levine 6-level classification scheme [17,20] (Table 1).

Echocardiographic examinations, with simultaneous electrocardiogram were performed by a board-certified cardiologist (A.F. and S.S.) or a cardiology resident (J.P.R.) under direct supervision using the same portable ultrasound machine (MyLab 30 Gold VET, Esaote, Florence, Italy) equipped with a phased array 2.5–5.0 MHz transducer. No dogs were sedated. The echocardiographic examinations were performed according to the recommendations for standard transthoracic echocardiography in the dog [21]. Colour Doppler was used to check for any valvular regurgitation or any other abnormalities in blood flow. Echocardiographic loops were recorded and saved for off-line analysis.

The diagnosis of MMVD was defined as the presence of MR on colour Doppler mode, in addition to thickened leaflets and/or mitral valve prolapse (MVP). The mitral valve thickness was visually assessed by 2D echocardiography [7] from both right parasternal and left apical views. MVP was defined as an abnormal systolic protrusion of mitral valve leaflets into the left atrium [22], which was evaluated using the right parasternal long axis 4-chamber view and classified as absent, mild, or severe according to the standardized method previously described [18,23]. M-mode was applied on the right parasternal short-axis view at the level of the papillary muscles for assessment of the LV internal diameters in diastole (LVIDd) and systole (LVIDs). The LV diameters were normalized to BW according to the following formulas: LVIDd normalized (LVIDdN) = LVIDd (cm)/(BW [kg])^0.294^ and LVIDs normalized (LVIDsN) = LVIDs (cm)/(BW [kg])^0.315^ [24]. The LA and aorta (Ao) diameters were measured from the right parasternal short-axis view at the level of the Ao aligned to the commissure of the aortic non-coronary and left coronary cusps at early ventricular diastole as previously described, without including the pulmonary veins [25]. Left atrium to Ao ratio (LA/Ao) was calculated and recorded. Cardiac remodelling was defined as echocardiographic findings of LA and/or LV enlargement secondary to chronic MMVD. The LA was considered dilated if the LA/Ao ≥ 1.6 [4]. The LV was considered dilated in diastole when LVIDdN was ≥1.73, and in systole when LVIDsN was ≥1.14 [4,24]. In order to stratify the degree of cardiac remodelling, a scoring system using the 3 markers of cardiac enlargement studied (LA/Ao, LVIDdN and LVIDsN) was arbitrarily generated. Individuals were scored as 0/3, 1/3, 2/3 and 3/3 when they had none, one (LVIDdN or LA/Ao), two (LVIDdN and LA/Ao) or three (LVIDdN, LA/Ao and LVIDsN) markers of cardiac remodelling above the established limits, respectively. The criteria for remodelling are outlined in Table 2.

In the presence of tricuspid regurgitation (TR), continuous wave Doppler interrogation was attempted, and it was archived if the resultant spectral trace was considered adequate for estimation of the systolic pulmonary arterial pressure. Pulmonary arterial pressure was estimated solely by measuring the TR velocity. Pulmonary hypertension was assessed using three different cut-offs: ≥2.8 m/s [26,27], >3 m/s [10,28], and >3.4 m/s [29].

All measurements were calculated from the average of 3 cycles, and from these data the extent of remodelling was determined. A single operator, who was blinded to the details of the dog, measured all the echocardiographic variables.

Dogs with significant concurrent systemic disease or cardiac diseases other than MMVD with or without TR, incomplete echocardiographic examination, or persistent arrhythmias, considered to affect the quality of the echocardiographic examination, were excluded from the analysis.

### Data and Statistical Analysis

An ad hoc electronic form was used to collect all study variables. Qualitative variables were described with absolute and relative (percentage) frequencies, whereas quantitative variables were summarized with means (standard deviations, SD) or medians (interquartile ranges, IQR) in the case of parametric and non-parametric distribution, respectively. The Shapiro–Wilk normality test was used to assess the distribution of the quantitative variables. Spearman’s rank correlation test was used to measure the degree of association between age and murmur intensity. The correlation coefficient (rho) was interpreted as previously described [30]. Logistic regression analysis was performed to assess the relationship between the predictive variables age, sex, and BW, and the categorical variable remodelling (presence or absence), remodelling grade (0/3, 1/3, 2/3, 3/3), MVP (presence or absence), MVP severity (absent, mild and severe), and PH (presence or absence) for all the 3 selected TR velocity cut-offs values. A two-tailed *p*-value less than 0.05 was considered statistically significant. The statistical software STATA version 16 (StataCorp, StataCorp, College Station, TX, USA) was used to perform statistical computations.

## 3. Results

One hundred and twenty-eight CKCSs eight years of age or older, belonging to breeders and private owners, were presented for examination. One dog was excluded because of a congenital heart disease, and another because of incomplete echocardiographic examination due to a lack of cooperation, leaving 126 CKCSs included in the analysis.

The median age at the time of examination was 10.3 years (IQR 9.2–12). The age distribution is shown in Table 3. Median BW was 8.2 kg (IQR 7.2–9.8). There were 44 male dogs (35%), of which 27 (21.5%) were entire and 17 (13.5%) were neutered. Female dogs represented 65% (*n* = 82) of the population, with 27 (21%) being entire and 55 (44%) neutered.

Presence of clinical signs was reported in 24 dogs (19%). Owner-reported clinical signs were cough (*n* = 21; 16.7%) and exercise intolerance (*n* = 9; 7.1%). At the time of examination, 14 dogs (11.1%) diagnosed with MMVD by their own veterinary surgeon received at least one treatment as follows: pimobendan (*n* = 13; 93%), furosemide (*n* = 7; 50%), an ACE inhibitor (*n* = 3; 21.5%), and spironolactone (*n* = 1; 7%). On physical examination, dyspnoea was present in five dogs (4%), and ascites in two (1.6%).

**Figure 1:**
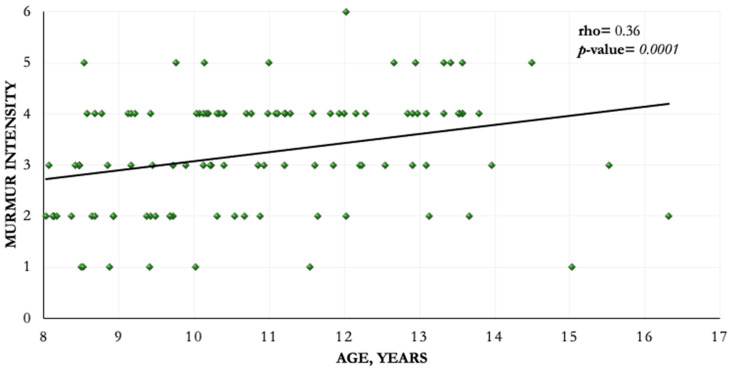
Correlation between murmur intensity and age.

A systolic murmur was detected in 112 dogs (89%), 11 dogs (9%) did not present a murmur, and in 3 dogs (2%) the presence or absence of a murmur was not recorded. The murmur intensity and point of maximum intensity (PMI) was recorded in 109 of 112 dogs with murmurs. The PMI was localized on the left apical area in 104 patients (93%) and on the right heart area in five dogs (4%). Intensity of left apical murmurs was scored as grade 1 (*n* = 7; 6%), grade 2 (*n* = 24; 21%), grade 3 (*n* = 26; 23%), grade 4 (*n* = 37; 33%), grade 5 (*n* = 9; 8%), and grade 6 (*n* = 1; 1%). Right heart murmur intensities were scored as grade 2 (*n* = 1; 1%), grade 4 (*n* = 3; 3%) and grade 5 (*n* = 1; 1%). During auscultation, the presence of irregular heart rhythm was detected in 16 dogs (12.7%). Mean (SD) heart rate was 126 (21) bpm; respiratory rate was recorded in 111 dogs, and the mean (SD) RR was 28 (7) bpm. A fairly positive significant correlation was found between murmur intensity and age (rho = 0.36; *p* = 0.0001). This linear relationship is represented in Figure 1.

On echocardiographic examination. all 126 CKCSs (100%) were diagnosed with MMVD; 123 of them (98%) had both MR and TR. Mitral valve prolapse was evidenced in 111 dogs (88%); classified as mild in 91 (82%) and severe in 20 (18%) dogs. Fifty-six dogs (44.4%) had heart chamber sizes within normal limits, and in three of these, the owners reported clinical signs commonly seen with MMVD (cough *n* = 2, exercise intolerance *n* = 1), with one dog being treated with pimobendan. Dogs without echocardiographic signs of cardiac remodelling had a median (IQR) age of 9.7 (8.8–10.9); within them, 39 (70%) were 8–10 years old, 13 (23%) were 11–13 years old, and 4 (7%) were >13 years old (Table 3). Nine out of 56 dogs (16%) without cardiac remodelling did not have murmur on cardiac auscultation.

Cardiac remodelling was present in 70 (55.6%) dogs. In 21 of them (30%), the owners reported clinical signs commonly associated with their disease, including cough and exercise intolerance in six of them (8.6%), only cough in thirteen (18.6%), and only exercise intolerance in two (2.8%). Ten clinically affected CKCSs with cardiac remodelling were under cardiac therapy. Dogs with echocardiographic signs of cardiac remodelling had a median (IQR) age of 11 (9.7–12.3). As shown in Table 3, the majority of the dogs with remodelling were less than 13 years old. Within them, 30 were 8–10 years old (43.5% of dogs of this age), 34 were 11–13 years old (72.3% of dogs of this age), and 6 were >13 years old (60% of dogs of this age). In the cardiac remodelling group, 66 dogs (94% of dogs with cardiac remodelling) had LVIDdN values ≥ 1.73, 38 (54% of dogs with cardiac remodelling) had LA/Ao ≥ 1.6, and 13 (18.6% of dogs with cardiac remodelling) had LVIDsN ≥ 1.14. The results of the cardiac remodelling classifications are showed in Table 4. Two dogs out of 70 (3% of dogs with cardiac remodelling) did not present murmur on auscultation. Both of them scored 1/3 on the cardiac remodelling classification.

Nineteen dogs (17.6%) had TR velocity ≥ 2.8 m/s, of which 10 (9.3%) had TR velocity > 3 m/s and 5 (4%) had TR velocity > 3.4 m/s.

During echocardiographic examinations, arrhythmic events were observed in 11 dogs, and included supraventricular premature complexes (*n* = 6; 4.8%), second degree atrioventricular block (*n* = 3; 2.4%), ventricular premature complexes (*n* = 1; 0.8%), and sinus arrest (*n* = 1; 0.8%).

The logistic regression analysis showed that gender did not have a significant effect on remodelling (presence or absence), remodelling grade (0/3, 1/3, 2/3, 3/3), MVP (presence or absence), MVP severity, or PH (presence or absence). Body weight did not have a significant effect on remodelling (presence or absence), remodelling grade (0/3, 1/3, 2/3, 3/3), or MVP (presence or absence), but it was negatively associated with MVP severity (OR 0.8, 95% CI: 0.6–0.99, *p* = 0.04). Age was significantly associated with the presence of remodelling (OR 1.3, 95% CI: 1.0–1.6, *p* = 0.01), with the remodelling grade (OR 1.27, 95% CI: 1.1–1.5, *p* = 0.01), with the MVP severity (OR 1.3, 95% CI: 1.1–1.7, *p* = 0.009) and with the presence of PH when it was defined as TR velocity > 2.8 m/s (OR 1.5, 95% CI: 1.2–2, *p* = 0.003). In contrast, when PH was defined as TR velocity > 3 and 3.4 m/s, the effect of age on it was not statistically significant (*p* > 0.05). Age did not exert a significant effect on MVP presence.

## 4. Discussion

We initially hypothesised that within the elderly CKCS population, there is a sub-cohort of MMVD-affected dogs that do not have cardiac remodelling. Our results showed that the prevalence of MMVD in a population of 126 CKCSs ≥ 8 years old was 100%. Despite the expected high prevalence of MMVD in our elderly CKCS population [13,14,15,16,18,19,31], 56 dogs (44.4%) were clear of echocardiographic signs of cardiac remodelling which, based on the most recent American College of Veterinary Internal Medicine (ACVIM) Consensus Statement, would be classified as stage B1 MMVD [4]. Similar findings were reported in a previous study in which 38.5% of CKCSs aged over 10 years old, with echocardiographic evidence of MMVD, did not have LA or LV dilation [19]. These findings showed that a proportion of elderly CKCSs with confirmed MMVD do not have cardiac remodelling, which confirms the study hypothesis.

There were three dogs without cardiac remodelling where the owners had reported the presence of clinical signs commonly associated with MMVD, such as cough and exercise intolerance. Probably, these clinical signs, given the age of the subjects, were due to concomitant chronic respiratory and osteoarticular diseases rather than to MMVD.

Chronic and haemodynamically significant MMVD results in volume overload, which leads to LA and LVIDd enlargement. Later, as systolic function becomes impaired, LVIDs also increases [32]. In our CKCS population, a grading system was used to characterize echocardiographic remodelling (Table 2). Our results showed that the presence and the degree of cardiac remodelling were both significantly affected by age; indeed, for each year of age, the probability of having remodelling or having a more advanced grade of cardiac remodelling increased by about 30%. This result supports previous observations that age has a marked effect on MMVD progression and severity [18,19,22,32,33,34].

At physical examination, we found a left heart systolic murmur in 104 (82.5%) CKCSs ≥ 8 years old; moreover, our results evidenced that murmur intensity was significantly correlated with age. These findings are comparable with those obtained from previous studies and highlight, once again, the relationship between age and MMVD severity in CKCSs [14,16,17,18,19].

Murmur gradation can be simplified by classifying grades 1 and 2 as “low intensity”, grades 3 and 4 as “medium intensity”, and grades 5 and 6 as “high intensity” [17]. Based on this classification, the intensity of murmurs auscultated over the mitral area in our CKCS population could be defined as low in 30% (*n* = 31), medium in 61% (*n* = 63), and high in 9% (*n* = 10) of dogs. These numbers differ from those previously reported on a CKCS population of mean age 7.6 ± 2.6 years, in which there were more “high intensity” (41%) and fewer “medium intensity” left apical systolic murmurs (32%) [17]. This difference could be due to several reasons: firstly, the different age distribution of CKCSs included in this Haggstrom study (range 1.7 to 13.4 years) compared to our CKCS population (range 8 to 16.3 years); secondly, the two studies were conducted in two different European areas with different CKCS populations: and finally, the Haggstrom study was performed more than 20 years before our study, therefore the selection programs implemented by CKCS clubs may have led to a reduction in disease severity in CKCSs [35,36]. An interesting finding was that 16% of the 56 dogs with echocardiographic evidence of MMVD and without signs of cardiac remodelling did not present murmur on cardiac auscultation. This percentage is lower than that reported in a recent publication [31], but it should be taken into account when applying breed schemes based solely on auscultation [36].

On echocardiographic examination, MVP was a common finding in our population (88%). This result is in accordance with previous studies conducted on CKCSs [16,18,31]. Our results showed that the presence and the severity of MVP were independent of sex; on the contrary, MVP severity was significantly positively associated with age and negatively with BW. A one-year increase in age was associated with a 30% increase in the risk of increasing MVP severity; conversely, a one unit increase in dog’s BW was associated with a 20% decrease in the risk of increasing MVP severity. These results are in agreement with what was previously reported in CKCSs [18].

During echocardiographic examination, we observed arrhythmic events in 11 dogs (8.7%). In the veterinary literature, variable percentages of arrhythmias in canine MMVD are reported [34,37,38,39]. This high variability of results is probably due to the different inclusion criteria used to select patients, to the different severity stages of the disease, to the different diagnostic modalities, and to the different CKCS populations used in these studies.

In the present study, 98% of dogs were affected by both MR and TR. A high prevalence of degenerative tricuspid valve lesions (90%) has previously been described in older dogs with degenerative mitral valve disease based on pathology [6], although the echocardiographic evidence of TR is a common occurrence even in healthy CKCSs [40].

Pulmonary hypertension is commonly associated with MMVD in dogs. It is probably due to a combination of pulmonary venous hypertension and pulmonary arterial vasoconstriction and remodelling associated with chronic hypoxia [29,41]. Considering the TR velocity cut-off of 2.8 m/s, the prevalence of PH in our population of CKCSs affected by MMVD was 17.6%, and age significantly affected the presence of PH; indeed, for each year of age, the likelihood of having PH increased by about 50%. The prevalence of PH in our CKCS population was significantly lower compared to that obtained in a previous study conducted on CKCSs of 9.72 ± 2.26 years old (75.5%), in which the authors considered the same TR cut-off value of 2.8 m/s [27]. This different result could be due to the different inclusion criteria used in this study by Sudunagunta et al., in which only dogs with ACVIM stage B2 or higher MMVD were included. Another possible explanation could be that our population, being made up exclusively of elderly subjects, did not include subjects with more severe pathology, who died at a young age. The TR velocity cut-off of 2.8 m/s, commonly used to define PH in dogs, has recently been questioned by the ACVIM guidelines, which recommend the use of a cut-off of 3.4 m/s when diagnosing PH based solely on TR velocity measurements [29]. Our results showed that when using cut-offs of 3 m/s and 3.4 m/s, the prevalence of PH was considerably reduced to values of 9.3% and 4%, respectively, and age, in both cases, was not significantly associated with the presence of PH. PH is a parameter of considerable clinical and prognostic importance in dogs with MMVD [10]; therefore, we can state that in our elderly CKCS population affected by MMVD, the prevalence of PH was very low, and thus it did not represent an element of concern.

Our population comprised a higher proportion of female dogs (65%). This could be because some of the dogs included in the study were brought by breeders, and it is not unusual for them to keep more females than males in their populations. A second hypothesis could be that because male CKCSs develop MMVD at a younger age than female dogs [33], a lower percentage of them reach an older age. The participation in this study was based on a call for volunteers through many different CKCS clubs. It is difficult to be certain about the influence of this voluntary participation and whether it could have introduced some selection bias, not representing a completely random population of aged CKCSs. We considered that this system would allow a more heterogeneous inclusion than assessing dogs admitted to a referral veterinary hospital or general practice, but this could be considered a limitation of the study.

Our study has some other limitations. First, the echocardiographic examination was restricted to certain views and measurements. This limited the information obtained, but in contrast, allowed for an increased number of studies to be performed, and broadening of the geographic locations where the cases were examined potentially decreased the homogeneity of the population. Another limitation of our study is the fact that some of the dogs included were taking cardiac medications that could affect haemodynamics, with a potential effect on some of the measurements acquired. The percentage of dogs taking this sort of medication was low (11%), and we considered that excluding these patients would have probably introduced an important selection bias towards dogs with early phases of the condition. Finally, a further limitation was the fact that dogs aged >13 years were underrepresented, which does not allow us to exclude the fact that our results could be influenced by the different sample size of the age categories.

## 5. Conclusions

This study showed that despite the 100% prevalence of MMVD in this population of aged CKCSs, a relatively large proportion (44%) showed an absence of cardiac remodelling, indicating that a proportion of elderly CKCSs with confirmed MMVD do not undergo advanced stages of this pathology. This could be because the form of MMVD affecting CKCS at a young age, for which strong heritability has been demonstrated [42], is more aggressive and leads to death in young subjects, while a sub-cohort of dogs are affected by milder disease, more similar to that affecting all elderly dogs regardless of breed [43], which allows them to live longer. Identification of genetic and pathophysiologic mechanisms that lead to delayed progression of the disease may warrant future studies in order to reduce the clinical impact of the condition in this breed.

## Figures and Tables

**Table 1:** Murmur grading scheme.

Grade	Definition
1	Very soft, audible after few minutes of auscultation
2	Soft murmur but readily detected after a few seconds
3	Moderate-intensity murmur
4	Loud murmur but not accompanied by a precordial thrill
5	Loud murmur accompanied by a precordial thrill
6	Very loud murmur that produces a palpable thrill still audible after stethoscope is removed from the chest

**Table 2:** Remodelling score classification.

Size of the Left Heart Chambers	Remodelling Score
LVIDdN < 1.73 and LA/Ao < 1.6 and LVIDsN < 1.14	0/3
LVIDdN ≥ 1.73 or La/Ao ≥ 1.6	1/3
LVIDdN ≥ 1.73 and La/Ao ≥ 1.6	2/3
LVIDdN ≥ 1.73 and LA/Ao ≥ 1.6 and LVIDsN ≥ 1.14	3/3

LVIDdN, left ventricular internal diameter in diastole normalized to body weight; LA/Ao, left atrium to Ao ratio; LVIDsN, left ventricular internal diameter in diastole normalized to body weight.

**Table 3:** Age distribution of dogs.

Dogs		Age Distribution, Years			
	*n* (%)	Median, (IQR)	8–10, *n* (%)	11–13, *n* (%)	>13, *n* (%)
CKCSs included in the analysis	126 (100)	10.3 (9.2–12)	69 (54.8) *	47 (37.3) *	10 (7.9) *
CKCSs with cardiac remodelling	70 (55.6) *	11 (9.7–12.3)	30 (43.5) **	34 (72.3) **	6 (60) **
CKCSs without cardiac remodelling	56 (44.4) *	9.7 (8.8–10.9)	39 (56.5) **	13 (27.7) **	4 (40) **

IQR, interquartile range; CKCS, Cavalier King Charles Spaniel. * percentage calculated on the total number of dogs included in the analysis (*n* = 126). ** percentage calculated on dogs of that age category.

**Table 4:** Results of the remodelling score classification.

Left Heart Chambers Size	Remodelling Score	Dogs, *n* (%)
LVIDdN < 1.73 and LA/Ao < 1.6 and LVIDsN < 1.14	0/3	56 (44.4) *
LVIDdN ≥ 1.73 or La/Ao ≥ 1.6	1/3	36 (51.4) **
LVIDdN ≥ 1.73 and La/Ao ≥ 1.6	2/3	28 (40) **
LVIDdN ≥ 1.73 and LA/Ao ≥ 1.6 and LVIDsN ≥ 1.14	3/3	6 (8.6) **

LVIDdN, left ventricular internal diameter in diastole normalized to body weight; LA/Ao, left atrium to Ao ratio; LVIDsN, left ventricular internal diameter in diastole normalized to body weight. * percentage calculated on the total number of dogs included in the analysis (*n* = 126) ** percentage calculated on dogs with cardiac remodelling.

## Data Availability

Data are available on request to the authors.
